# Anesthesia isn’t sleep: The neuronal dynamics of immobility in isoflurane-anesthetized *C. elegans* differ from the activity patterns of previously established sleep-like quiescent states

**DOI:** 10.1371/journal.pone.0324323

**Published:** 2025-05-30

**Authors:** Andrew S. Chang, Laura Mazuera, Christopher V. Gabel, Christopher W. Connor

**Affiliations:** 1 Department of Physiology and Biophysics, Boston University School of Medicine, Boston, Massachusetts, United States of America; 2 Department of Anesthesiology, Peri-operative and Pain Medicine, Brigham and Women’s Hospital, Boston, Massachusetts, United States of America; 3 Boston University School of Medicine, Boston, Massachusetts, United States of America; 4 Department of Anesthesiology, Peri-operative and Pain Medicine, Brigham and Women’s Hospital, Boston, Massachusetts, United States of America; University of Nebraska Medical Center College of Medicine, UNITED STATES OF AMERICA

## Abstract

**Background:**

*C. elegans* possesses a precisely defined pattern of neuronal activation associated with its quiescent sleep-like state. As for higher-order creatures, this state includes the induction of atonia and immobility. In *C. elegans*, activation of the GABAergic neuron ALA directly inhibits AVE, a crucial command motor interneuron. By comparing this stereotypical pattern of activation to the activity seen within this neuronal circuit during anesthesia, we may determine if the atonia and immobility observed in anesthetized *C. elegans* is recruited via the same neuronal mechanism as in quiescence.

**Methods:**

Neuronal activity in *C. elegans*, as measured by fluorescence intensity of the calcium-indicator GCaMP6s, was captured using light-sheet microscopy under exposure to increasing depths of isoflurane anesthesia (n = 20). Neuronal identities were determined using the NeuroPAL nuclear labeling system. The activity of neurons in the sleep atonia pathway were identified for analysis. Neuronal traces were differentiated, and signal coherence between traces were calculated as Pearson correlation coefficients. Differences among conditions were assessed using ANOVA.

**Results:**

During quiescence, we observe the characteristically strong activation of ALA and RIS, with suppression of other neurons. In the awake animal, neuronal activity in ALA and AVE remains moderately negatively correlated (r = -0.286). However, isoflurane anesthesia in *C. elegans* does not result in an increase in the negative correlation between these two neurons: we observe a progressive loss and inversion of this baseline negative correlation, resulting in a significantly different positive correlation when deeply anesthetized (r = 0.229: p = 0.003, Type III ANOVA). In comparison, isoflurane anesthesia suppresses the positive correlation between AVE and a strongly-connected key member of the command motor interneuron circuit, AVA (p = 0.007, Type III ANOVA).

**Conclusions:**

Immobility is a fundamental characteristic of both anesthesia and naturally occurring sleep-states. However, we show that, in *C. elegans*, immobility in the anesthetized state is not produced by activating the innate neurological mechanism of quiescence.

## Introduction

In humans and other mammals, sleep paralysis is triggered by the activation of glutamatergic neurons in the sublaterodorsal nucleus, which in turn trigger neurons in the ventral medial medulla to release inhibitory GABA and glycine onto skeletal motoneurons [1,2]. Physiological sleep is accompanied by immobility and atonia of skeletal muscles. The loss of atonia during sleep, known as REM Sleep Behavior Disorder (RBD, ICD-10 CM: G47.52), is potentially an early harbinger of certain neurodegenerative disorders [3]. Famously, lesions of the dorsal pontine tegmentum in the cat lead to awake-like physical behavior during sleep because this inhibition is lost [4,5]. Similar inhibitory mechanisms of movement during sleep are present in lower animals too.

The occurrence of sleep in lower-order creatures such as drosophila (*D. melanogaster*), zebrafish (*D. rerio*) and *C. elegans* is determined by observed behavioral criteria. These creatures possess neuronal circuitry that produces physical immobility and reduced sensitivity to external stimuli (i.e., increased sensory thresholds) during these quiescent sleep-like states [6–8]. *C. elegans* demonstrate a quiescent state called lethargus that is equivalent to sleep [9] that is entered into at four developmentally-timed molting intervals. *C. elegans* also demonstrate periods of quiescence sleep-like states at other non-fixed times throughout its lifespan [10] that may be entered into for reasons of satiety (“satiety-induced quiescence”), starvation (“L1 arrest”), or stress recovery (“stress-induced sleep”), among others. Great progress has been made in *C. elegans* in understanding the neuronal signaling that leads both to the onset of quiescence and to concomitant immobility and atonia, enabled by the detailed characterization of this creature’s simple-but-effective nervous system [11]. Briefly, the onset of quiescence is marked by activation of the neuron RIS [12], which releases a FLP-11 neuropeptide [13] and also caused activation of the neuron ALA [14]. Almost all other *C. elegans* neurons become inactive during this time [15]. The activation of the ALA neuron leads to direct inhibitory GABAergic signaling onto the bilaterally symmetric neurons AVEL and AVER [16], which form an essential part of the *C. elegans* interneuron locomotor control circuit [17]. Thus, *C. elegans* has a well-characterized circuit for inducible immobility dependent on activation of key sleep-associated neurons ALA and RIS, which facilitates atonia in quiescent sleep-like states including both developmental lethargus [15] and stress-related quiescence which occurs throughout all life stages of the animal [10,18].

Immobility is a component of the general anesthetic triad, though the mechanisms by which volatile anesthetics produce immobility are less certain. Both sleep and the anesthetic response to volatile agents are highly-conserved evolutionary behaviors. A reasonable question is thus whether the action of volatile anesthetics produce atonia by recruiting the neurological mechanisms of physiological sleep states. C. *elegans* is well-established as a model system for volatile anesthetics, in terms of physiological behavior [19] and in the characterization of changes in the activity and patterns of communication within its nervous system [20]. *C. elegans* present unprecedented capabilities for “whole brain” imaging with signal cell resolution not possible in higher organisms. We therefore examine the behavior of the sleep atonia pathway under isoflurane anesthesia to determine whether its activity is physiologically consistent with sleep or not.

## Materials and methods

### *C. elegans* strains

**Fig 1:**
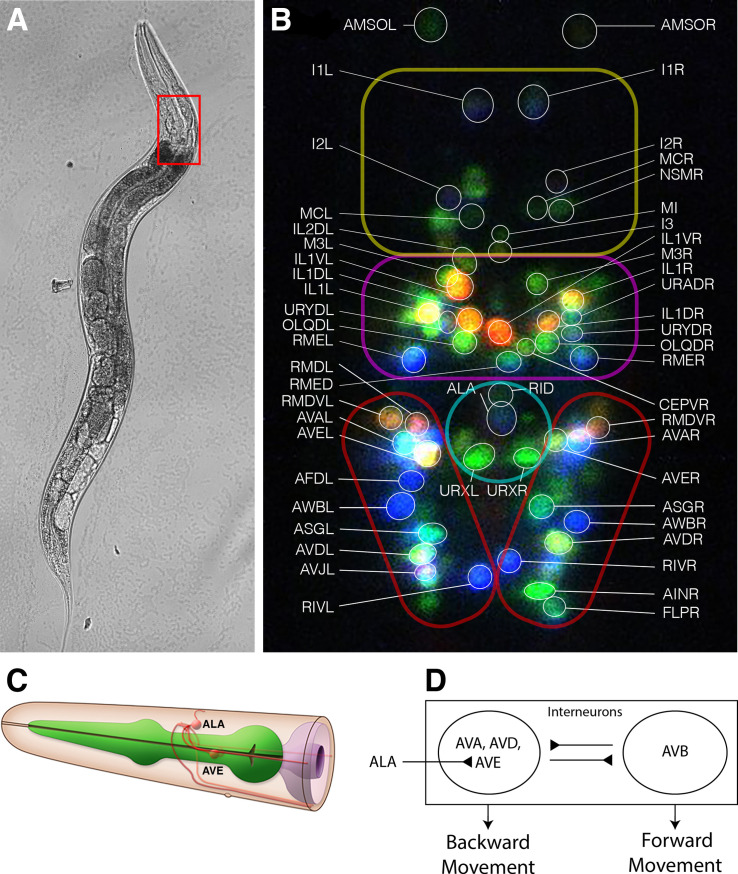
Gross and functional neuroanatomy of Caenorhabditis elegans. (A) *C. elegans*, standard bright field microscopy. The red box delineates the region of the head that contains the primitive *C. elegans* brain. (B) Maximum Intensity Projection superposition of seventeen 1 µm slices through the *C. elegans* brain, showing the identification of many neurons using the NeuroPAL identification scheme. A false-color representation is used in which expression of mTagBFP2 is shown in blue, CyOFP1 is shown in green, and mNeptune2.5 is shown in red. The enclosures delineate the *C. elegans* ganglia: the anterior pharyngeal bulb is marked in yellow; the anterior ganglion is marked in magenta; the dorsal ganglion is marked in turquoise and contains the neuron ALA (blue); the lateral ganglia are marked in scarlet and contain the neurons AVA (cyan) and AVE (almond). The ventral ganglion, which is located more caudally and contains the unilateral neuron RIS on the right side, is not shown. (C) Composite neuroanatomic representation of the ALA and AVE neurons, showing the cell bodies and axonal processes. Modified with permission from WormAtlas. [22] (D) Functional schematic of the *C. elegans* locomotor interneuron control circuitry, including inhibitory control of AVE by ALA.

*C. elegans* were cultivated following standard procedures, maintained at 20°C on Nematode Growth Media agar seeded with *E. coli* OP50 as a food source. Imaging experiments were performed using young adult hermaphrodites, modified to express fluorophores in neurons. All neurons express a green calcium-sensitive fluorophore GCaMP6s in the soma for reporting activity and a fixed red fluorescent fluorophore RFP in the nucleus for tracking (*zfIs124[Prgef-1::GCaMP6s]; otIs355[Prab-3::NLS::tagRFP]*, available as transgenic strain QW1217). Neurons also variably expressed three additional fixed fluorophores in their nuclei (blue mTagBFP2, orange CyOFP1, and far-red mNeptune2.5) under a selection of promoters as described in the *C. elegans* neuronal identification system NeuroPAL, available as transgenic strain OH15263 [21]. A representative example of *C. elegans* is shown in Fig 1A.

### Imaging of the quiescent sleep-like state

Although the physiology of sleep in *C. elegans* is most established in the intervals around lethargus [9], we know that *C. elegans* can enter a sleep state during most conditions and stages of their life [10]. Furthermore, we know that *C. elegans*, when enclosed in a microfluidic environment, will spontaneously enter brief intervals of quiescence of a few minutes duration and that these quiescent periods satisfy the four criteria of sleep in *C. elegans*: stereotypical posture, reversibility, decreased response to stimuli, and homeostasis [23]. We have shown that *C. elegans* enter similar quiescent states when encapsulated for light-sheet microscopy imaging, and that the likelihood of entering into a quiescent state increases with the animal’s age. By classifying global neural quiescence as time points in which at least 70% of neurons in an animal were inactive, we previously determined that adult day 1 worms display global quiescence 1% of the time whereas, on average, day 9 worms do so 9% of the time [24].

A total of five *C.elegans* aged to 9 days were encapsulated and imaged in an unanesthetized state for periods of 10 minutes [24]. *C. elegans* at this age frequently enter brief episodes of quiescence when undisturbed. Imaging of neurological activity was performed with nuclear RFP for localization and somatic GCaMP6s for activity, as above, until a suitable example of entry and exit from quiescence was observed.

### Anesthesia, imaging, acquisition of activity, and neuron identification

A total of n = 20 *C. elegans* were anesthetized and imaged individually. Specimens were encapsulated in a permeable hydrogel and then exposed to isoflurane equilibrated to concentrations equivalent to 0%, 2%, 4% and 8%, using protocols as defined earlier [25]. These isoflurane concentrations correspond to MAC values of approximately 0, 0.66, 1.33 and 2.66 in *C. elegans*, and we have shown that exposure to isoflurane 8% induces complete behavioral immobility and unresponsiveness to noxious stimulus [26]. Imaging in 3D was performed using a Dual Inverted Selective Plane Illumination (DISPIM) microscope (Applied Scientific Instrumentation, USA) at 2 volumes/second, as according to previous protocols. Briefly, a 488 nm laser at 5 mW excites the GCaMP6s in the soma of the neurons, but the degree of green fluorescence of this reporter is dependent on the intracellular calcium concentration. The activity of each neuron can therefore be captured optically, non-invasively, and *in vivo*. A 561 nm laser at 20 mW excites the red nuclear RFP, allowing for any motion of neurons within the imaging frame to be tracked and compensated. Each imaging sequence lasts for 10 minutes. Before and after each image sequence, volumetric images are obtained with laser illumination at 405 nm at 40 mW, 488 nm at 20 mW and 639 nm at 100 mW which serve to excite the identification fluorophores mTagBFP2, CyOFP1 and mNeptune2.5 respectively. Bandpass filters are used at the imaging camera on the emitted signals in order to eliminate crosstalk between these fluorophores [21].

Animals were imaged at a rate of two volumes/second, with a voxel size of 0.1625 μm × 0.1625 μm × 1 μm, capturing both nuclear-RFP and cytoplasmic-GCaMP6s fluorescence. Using the methods described in Awal *et al.* [25] and Wirak *et al.* [24], we tracked track 120 RFP-labeled nuclei per animal in three dimensions. Briefly, the centerpoints of each nucleus were identified by convolving the RFP channel data with a Laplacian-of-Gaussian function. Matching of neurons from one timestep to another was performed using the Kuhn–Munkres Assignment algorithm, always selecting the match that best minimizes the total squared distance between neuron positions. The identified locations of the nuclei were error-corrected using smoothing and consensus based on the location of the surrounding neurons [27]. We then extracted GCaMP6s signals from the surrounding soma for each tracked neuronal nucleus. Normalized fluorescence (ΔF/F_0_) was calculated for all measured neuronal activity traces. For each neuron, F_0_ was calculated as the mean value of the lowest 1% of measurements made for that neuron.

Fig 1B shows an example of neuron identification in the *C. elegans* head. Neurons are identified by a combination of their known anatomic relationships and also by their nuclear color using the NeuroPAL identification fluorophores. For example, ALA, located midline in the dorsal ganglion, expresses the mTagBFP2 identification fluorophore and thus appears blue. The bilaterally-symmetric locomotor interneurons AVEL and AVER (hereafter, collectively referred to as AVE) are located in the lateral ganglia and express the CyOFP1 and mNeptune2.5 fluorophores, and appear approximately almond-colored. The major command motor interneurons AVAL and AVAR (hereafter, AVA) are typically located anteriorly and laterally to AVE, additionally express mTagBFP2 and appear cyan. The ventral ganglion is not shown in this image, but is located more caudally and contains the unilateral, unpaired RIS neuron on the right side, colored pale green. Once all neurons of interest have been identified and tracked, their neuronal activity can be retrieved from the somatic green GCaMP signal.

Fig 1C shows a schematic of the anatomic positioning of ALA and the AVE neurons, including their major axonal processes. The caudal axonal processes of ALA pass proximately to the bilateral AVE cell bodies. Fig 1D shows a simplified block schematic of the mutually inhibitory behavior of the backward-movement and forward-movement command interneurons, including the GABAergic inhibitory action of ALA upon AVE in quiescing this circuit.

### Statistical methods

Images of *C. elegans* are acquired volumetrically (i.e., in three dimensions), but are shown in projection for illustration. We generate projections using a Maximum Intensity Projection (MIP) algorithm, in which each pixel brightness is assigned by the brightest voxel along that ray. This type of image projection is commonly used in clinical practice for viewing positron emission tomography images [28].

In order to analyze the relative activity of, for example, the ALA and AVE neurons under anesthesia, it is necessary that at least the ALA neuron and at least one of the two bilateral AVE neurons be identifiable in the imaged animal. It is not necessarily the case that all neurons will always be identifiable, and thus data may not always be obtainable. Indeed, it is expected when using labeling and signal extraction with NeuroPAL, that a variable subset of neurons and pathways will be identifiable in each experimental specimen [29].

Regularized numerical differentiation was performed using Chartrand’s algorithm [30]. Regularization in time-differentiation greatly reduces the effect of noise on the rate-of-change signal. Correlations were measured using the Pearson correlation coefficient. Distributions were initially compared with ANOVA for unbalanced datasets to determine whether at least any one of the condition means was statistically significantly different from the rest, with a standard value of *α* = 0.05. Subsequent pairwise comparisons were performed by obtaining the *p* value for Fisher’s F-statistic for the two distributions in question. As there are four observed levels of anesthesia (i.e., isoflurane at 0%, 2%, 4% and 8%), there are six pairwise comparisons possible (i.e., 0% v 2%, 0% v 4%, 0% v 8%, 2% v 4%, 2% v 8%, 4% v 8%). In order to claim statistical significance in a pairwise comparison, we apply the most conservative Bonferroni correction and require that *p* be less than *α* = 0.05/ 6.

## Results

**Fig 2:**
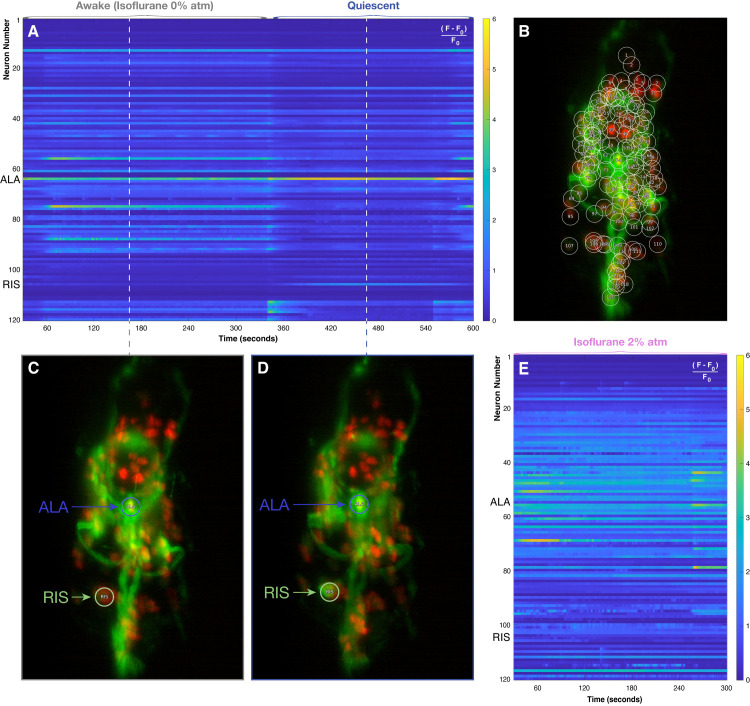
Neurological activity in *C. elegans* around an episode of lethargus, as compared to isoflurane anesthesia. (A) A heatmap of neuronal activity across 120 neurons in the head of an exemplar *C. elegans* at age 9 days in which an episode of sleep-like quiescence was captured. From around 60 seconds to 340 seconds, normal patterns of neuronal activity are observed. However, from 345 seconds to 550 seconds, neuronal activity becomes dramatically diminished with the exception of only two strongly active neurons, ALA and RIS. (B) Individual nuclei are marked red with RFP. The brightest 120 nuclei are automatically tracked and numbered in nose-to-tail order. Neuronal activity is reported by the green calcium-sensitive fluorophore GCaMP6s, expressed in the soma of the neurons. The image is a Maximum Intensity Projection. In this dataset, ALA is neuron #64 and RIS is neuron #106. (C) Snapshot of functional fluorescent activity in the awake state at time 165 seconds. ALA and RIS are specifically highlighted. The image is oriented like a plain film X-ray: nose-to-tail runs from top to bottom, and the right side of the animal is on the left side of the image. (D) Snapshot of functional fluorescent activity in the quiescent state at time 465 seconds, as for (C). RIS is now activated, ALA is hyperactivated, and other neurons have become relatively silent. (E) A heatmap of neuronal activity across 120 neurons in a different *C. elegans* under anesthesia with isoflurane 2%atm. The pattern of activity under anesthesia is qualitatively different from both the awake and quiescent states. In this particular imaging sequence, ALA is neuron #55 and RIS is neuron #105. Some variation in numbering is expected from worm to worm due to anatomy and physical orientation.

Fig 2A shows a heatmap of tracked neuronal activity in a senescent (day 9) *C. elegans* specimen, encapsulated and immobilized in a hydrogel for imaging but otherwise unanesthetized. Under these conditions, elderly *C. elegans* will frequently enter and exit brief bouts of sleep-like quiescence if left undisturbed, and the captured tracing here is characteristic of that behavior. Fig 2B shows one of the 3D time-sequence images of this *C. elegans*, including annotation of the 120 neurons detected, tracked and extracted during this imaging sequence. Entry into quiescence demonstrates a general silencing of neuronal activity with the exception of the unpaired neurons RIS and ALA. Normal spontaneous neurological activity resumes upon exiting quiescence. Figs 2C and 2D show snapshots of the activity within the head of the specimen during an awake period and during quiescence, respectively. The activation of the RIS neuron is obvious, along with the hyperactivation of ALA. These observations agree with established findings that demonstrate ALA and RIS activation and suppression of all other neurons during quiescence [15,23]. For comparison, Fig 2E shows an equivalent extraction of a short sequence of neuronal activity in a different *C. elegans* anesthetized by exposure to isoflurane 2%atm. It is apparent that the organization of the overall neuronal activity across the *C. elegans* head is qualitatively different in the anesthetized state compared to both the awake state and the quiescent state. However, this global observation is not sufficient to determine whether or not particular pathways for atonia are commonly activated in these states.

Table 1 describes the observability of the targeted neurons in this pathway over the n = 20 specimens, and identifies those imaging sequences which produced adequate observations, as per the Statistical Methods.

**Table 1:**
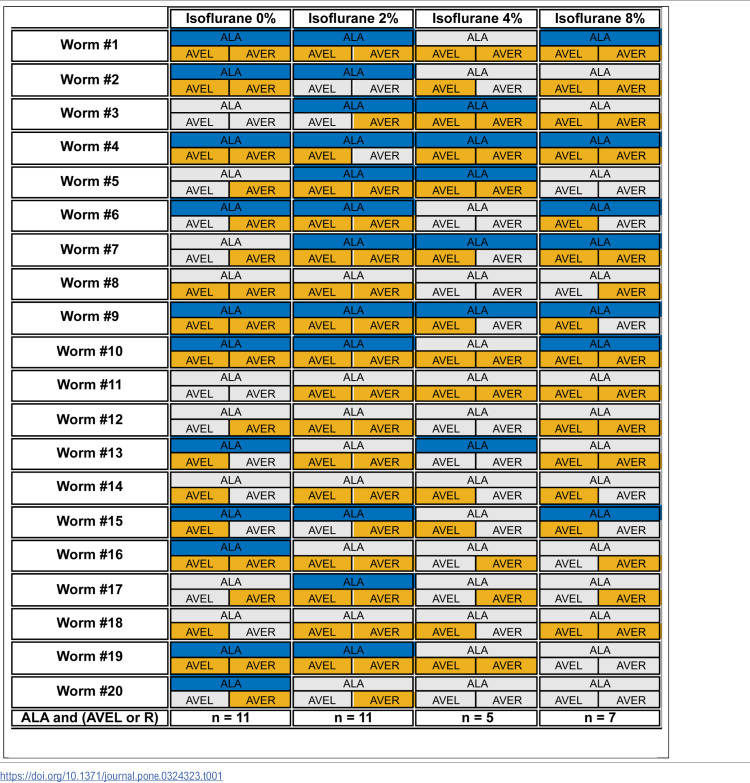
Experimental acquisition of activity data by subject number, isoflurane exposure, and neuron. Due to variations in expression of fluorophores between individual specimens, positioning of the specimen, physical compression of the ganglia, and movement at the microscopic level, complete identification and tracking of the neurons of interest may not necessarily be attainable. To contribute to the analysis of of the ALA-AVE immobility pathway, the ALA neuron plus at least one of the AVEL or AVER neurons must be obtainable. Where obtained, ALA and AVE are highlighted with their respective color (blue, almond), otherwise grey.

**Fig 3:**
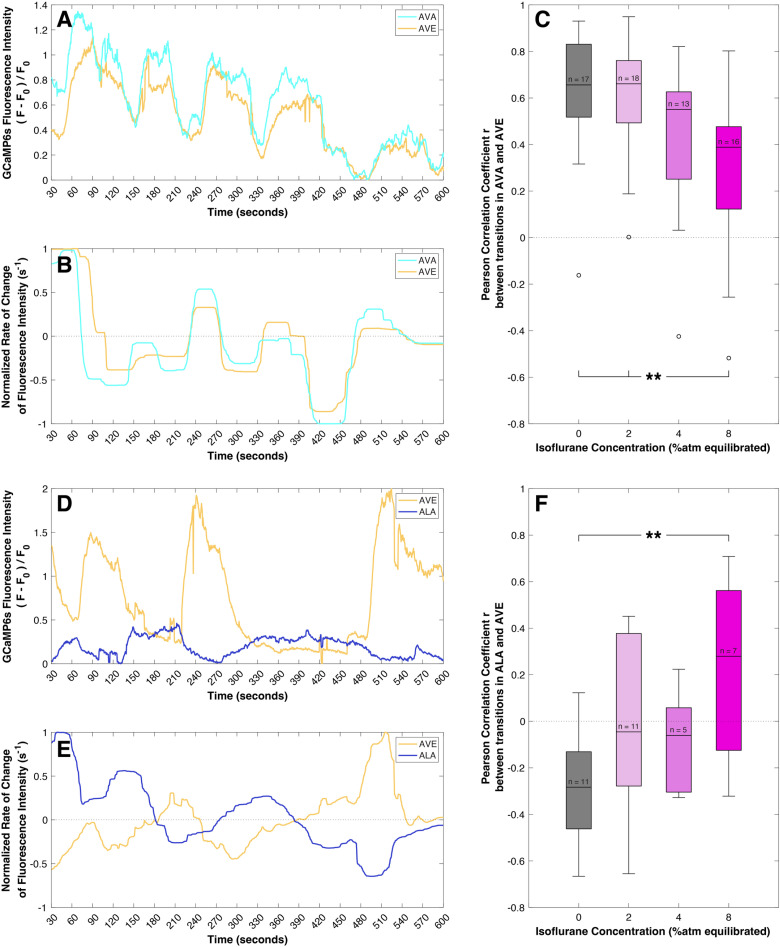
Activity and correlations between the neurons AVA and AVE, and between ALA and AVE under progressively increasing isoflurane anesthesia. (A) Exemplar activity of the AVA and AVE neurons in an unanesthetized *C. elegans* (i.e., isoflurane 0%). The GCaMP6s fluorescence, which fluctuates with activity, is normalized against the baseline brightness for each neuron. (B) Rate of change of fluorescent activity for these traces, calculated by regularized numerical differentiation and normalized to the range [-1,1]. Regularization greatly reduces the effect of noise on the rate-of-change signal. The strongly positively correlated relationship between the two neurons is clearly visible: as AVA changes positively or negatively, AVE follows. (C) Range of correlations between AVA and AVE under increasing levels of isoflurane exposure. Unanesthetized *C.elegans* is shown in gray (0%atm), and increasing levels of isoflurane exposure (2, 4, and 8%atm) are shown in increasingly vivid purple. Individual outliers are marked. Increasing isoflurane exposure weakens the strong positive coupling between these two neurons. (**, p < 0.01 and significant at the Bonferroni-corrected α = 0.05/ 6.) (D) Exemplar activity of the ALA and AVE neurons in an unanesthetized *C. elegans*, calculated as for (A). (E) Rate of change of fluorescent activity for these traces, calculated as for (B). The antagonistic, negative correlated relationship between these two neurons is clearly visible: as ALA more rapidly changes positively or negatively, AVE responds in the opposite sense. (F) Range of correlations between ALA and AVE under increasing levels of isoflurane exposure, calculated as for (C). Loss of negative correlation and increasing positive correlation between ALA and AVE are seen with increasing isoflurane exposure. (**, p < 0.01 and significant at the Bonferroni-corrected α = 0.05/ 6.).

**Fig 4:**
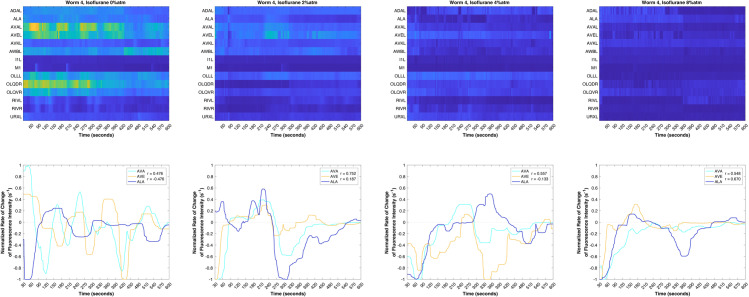
Activity and correlations of the neurons AVA, AVE and ALA in a single *C. elegans* under increasing amounts of isoflurane anesthesia. The overall fluorescent activity decreases as expected, but the correlation of the ALA and AVE pathway also becomes inverted relative to the unanesthetized state.

Fig 3A shows exemplar activity of the AVA and AVE neurons in an unanesthetized *C. elegans* (i.e., isoflurane 0%). The GCaMP6s fluorescence, which fluctuates with activity, is normalized against the baseline brightness for each neuron. The AVA and AVE neurons are known to be strongly interconnected and positively correlated in their action, as is obviously seen here. Since *C. elegans* neurons show graded rather than spiking activation, and also because differences exist in the degree of expression of GCaMP6s between neurons, it is easier to infer the functional relationship between neurons by examining how the change in activation of one neuron affects the change in activation of another. This time-differentiation also helps to exclude artifactual correlations between pairs of neurons that may occur simply if those neurons maintain relatively constant levels of activation, as we have done in previous studies [26]. Thus, we determine the effect of one neuron on another (i.e., the behavior of a pathway) by calculating the rate of change of fluorescent activity for these traces, obtained by regularized numerical differentiation and normalized to the range [-1,1], as shown in Fig 3B and as described in the Statistical Methods [26]. As is clearly visible, fluctuations in the activation of AVA lead comparable fluctuations in the activation of AVE in the awake, unanesthetized animal. Indeed, this is the expected and stereotypical behavior of the *C. elegans* motor circuit. Increasing levels of exposure to isoflurane, as shown in Fig 3C, lead to a progressive breakdown of the strong positive correlation between AVA and AVE, consistent with our previous studies on the motor circuit specifically and the *C. elegans* nervous system in general (*p* = 0.007, Type III ANOVA) [25,26]. In contrast, Figs 3D and 3E show the activation and relative fluctuations of ALA and AVE. In this case, an antagonistic, negative correlated relationship between the two neurons is clearly visible. Fig 3F shows the range of correlations observed between ALA and AVE under increasing levels of isoflurane exposure. A progressive loss of negative correlation and increasing positive correlation are seen with increasing isoflurane exposure, and a statistically significant difference in correlation is observed between the isoflurane 0% and isoflurane 8% conditions (*p* = 0.003, Type III ANOVA.) This change in the behavior of the ALA-AVE pathway with increasing isoflurane exposure is antithetical to the physiological behavior seen stereotypically in sleep/lethargus, in which ALA is activated while AVE activity is suppressed (*i.e.,* they are inherently anti-correlated). In Fig 4, we illustrate the activity in a number of identified neurons in a single *C. elegans* over a range of isoflurane levels, and show in detail how the relationships between AVA, AVE and ALA vary as the depth of anesthesia is progressively increased.

## Discussion

Sleep-like quiescent states in C. elegans are associated with activation of an atonia pathway in which increasing ALA activation causes inhibition of AVE and quiescence of the motor circuit [10,15,18]. The synaptic connection from ALA to AVE is GABAergic and inhibitory, and so the physiological behavior of these neurons is expected to be negatively correlated. As we see in Figs 3D and 3E, activation of ALA subsequently leads to the expected inhibition of AVE [16]. If the immobility caused by exposure to volatile anesthetic agents resulted from the recruiting of the physiological mechanism of atonia during lethargus or other sleep-like quiescent periods, then one would logically expect to see a strengthening of the appropriate correlation between the activity of the involved neurons with anesthesia, and then one would also expect the magnitude of that correlation to increase with increasing exposure to a representative agent such as isoflurane.

However, the results demonstrate an entirely different effect. While the Pearson correlation between ALA and AVE is indeed moderately negative in the awake worm (isoflurane 0%), as one would expect, increasing exposure to isoflurane causes the loss of inhibitory correlation in direct contrast to the known dynamics of these neurons during quiescence. Indeed, under exposure to isoflurane 8%, the correlation is effectively reversed when compared to the awake state (*r* = -0.286 at 0%, *r* = 0.229 at 8%, *p* < 0.005). At isoflurane 8%, the mild correlation of these neurons follows that of the nervous system as a whole (as we measured previously [25]) and may be due to generalized system effects and suppression of activity. Regardless, we must reject the hypothesis that volatile anesthetics recruit the native physiologic mechanism of sleep atonia in *C. elegans*, and we therefore conclude that these mechanisms of immobility are distinct.

We have previously demonstrated that isoflurane anesthesia in *C. elegans* is characterized by a suppression of mean positive correlation across the whole nervous system to low but non-zero levels [25]. However, when we isolate the behavior of specific pairs of synaptically linked neurons with correlated behavior at baseline, we see that some pairs of neurons (AVA-AVE) follow this pattern, while others (ALA-AVE) exhibit the opposite behavior: exposure to isoflurane results in seemingly increased signal correlation. How do we square this? When baseline correlation is positive (AVA-AVE), correlation shifts negatively under isoflurane anesthesia; when baseline correlation is negative (ALA-AVE), correlation shifts positively. Whether specifically inhibiting excitatory synapses or potentiating excitatory synapses, we may understand the networked behavior of isoflurane as producing a global weakening of communication between highly connected neurons [20]. This would be coherent with our previous finding of globally suppressed correlation since the large majority of synapses in the *C. elegans* connectome are excitatory, rather than inhibitory.

Neuronal identification with NeuroPAL in *C. elegans* provides an extremely flexible platform for dissection of neuronal behavior and networking. In this study, we describe a specific set of findings based on extraction of certain neuronal traces from a large dataset of neurons. Prior to the development of NeuroPAL, experiments of this scale were limited by the requirement to produce specific fluorescently-marked transgenic strains for each neuron type of interest. Freed from this limitation, large-scale investigations have begun to yield unexpected insights into the functional connectome [29]. The NeuroPAL labeling system in *C. elegans* was originally developed in order to measure gene expression with individual neuron resolution, requiring only a few static images that may be obtained with a confocal microscope [31]. In contrast, the use of calcium-sensitive fluorophores to obtain functional activity requires a long time-sequence of three-dimensional images to be reconciled so that individual neurons can be identified, tracked and their activity captured. This necessarily leads to gaps in acquisition and incomplete observations even in the most thoroughly performed studies [29,32,33]. However, in a sufficiently large dataset, it becomes possible to identify examples in which neuronal pathways of interest are identified (see Methods) and signal transduction can be observed [34].

Fig 4 illustrates the relationships and correlations between AVA and AVE and between AVE and ALA as captured in a single example animal across the range of isoflurane exposures. As indicated in Fig 1D and shown in Fig 3B, the activity relationship between the AVA and AVE neurons is generally well-correlated, especially when on the same laterality. The example shown in Fig 4 illustrates that this positive correlation is maintained between these two neurons even across the range of isoflurane exposures, despite the fact that activity as a whole is obviously diminished. In addition, Fig 4 illustrates how the absolute change in correlation in the ALA-AVE immobility pathway during isoflurane anesthesia is unusual, with a loss of anti-correlated activity with anesthesia. Ultimately, the effect of anesthesia on these neurons in this animal is opposite to that observed during quiescence, representing a concrete example of our conclusion that the immobility of isoflurane anesthesia and the immobility of sleep are qualitatively distinct phenomena in *C. elegans.*

### Disclosures

(Connor) Dr. Connor has consulted for Teleflex, LLC (Wayne, PA) on issues regarding airway management and device design and General Biophysics, LLC (Framingham, MA) on inhalational kinetics. These activities are unrelated to the material in this submission

## Supporting information

S1 Data Supporting Data. (ZIP)
